# External Mechanical Work in Runners With Unilateral Transfemoral Amputation

**DOI:** 10.3389/fbioe.2021.793651

**Published:** 2021-12-27

**Authors:** Hiroto Murata, Genki Hisano, Daisuke Ichimura, Hiroshi Takemura, Hiroaki Hobara

**Affiliations:** ^1^ Graduate School of Science and Technology, Tokyo University of Science, Chiba, Japan; ^2^ Artificial Intelligence Research Center, National Institute of Advanced Industrial Science and Technology, Tokyo, Japan; ^3^ Department of Systems and Control Engineering, Tokyo Institute of Technology, Tokyo, Japan; ^4^ Research Fellow of Japan Society for the Promotion of Science, Tokyo, Japan

**Keywords:** amputee locomotion, external mechanical work, bouncing gait, running-specific prosthesis, ground reaction forces

## Abstract

Carbon-fiber running-specific prostheses have enabled individuals with lower extremity amputation to run by providing a spring-like leg function in their affected limb. When individuals without amputation run at a constant speed on level ground, the net external mechanical work is zero at each step to maintain a symmetrical bouncing gait. Although the spring-like “bouncing step” using running-specific prostheses is considered a prerequisite for running, little is known about the underlying mechanisms for unilateral transfemoral amputees. The aim of this study was to investigate external mechanical work at different running speeds for unilateral transfemoral amputees wearing running-specific prostheses. Eight unilateral transfemoral amputees ran on a force-instrumented treadmill at a range of speeds (30, 40, 50, 60, 70, and 80% of the average speed of their 100-m personal records). We calculated the mechanical energy of the body center of mass (COM) by conducting a time-integration of the ground reaction forces in the sagittal plane. Then, the net external mechanical work was calculated as the difference between the mechanical energy at the initial and end of the stance phase. We found that the net external work in the affected limb tended to be greater than that in the unaffected limb across the six running speeds. Moreover, the net external work of the affected limb was found to be positive, while that of the unaffected limb was negative across the range of speeds. These results suggest that the COM of unilateral transfemoral amputees would be accelerated in the affected limb’s step and decelerated in the unaffected limb’s step at each bouncing step across different constant speeds. Therefore, unilateral transfemoral amputees with passive prostheses maintain their bouncing steps using a limb-specific strategy during running.

## 1 Introduction

Prevalence of carbon-fiber running-specific prostheses (RSPs) is one of the greatest progresses for Para athletics and contribute greatly to improve prosthetic user’s performance (Nolan, 2008; Tuakli-Wosornu et al., 2021). RSPs with energy storing capabilities have enabled individuals with lower extremity amputation to run by providing a spring-like leg function in their affected limb. Although the RSPs cannot generate mechanical power during the stance phase, mechanical testing has demonstrated a considerable (more than 90%) elastic energy return (Brüggemann et al., 2008; Beck et al., 2016). Human running is fundamentally described as a bouncing gait mechanism, where each lower limb behaves like a spring (Cavagna et al., 1964; Farley et al., 1993). The spring-like “bouncing step” using RSPs is considered a prerequisite for running; however, the principal characteristics of bouncing gaits using passive prostheses remain largely undetermined. In particular, unilateral transfemoral amputees (UTFAs) use various passive prosthetic components, such as RSPs, adapters, pylons, prosthetic knee joints, and sockets in the affected limb. Consequently, running is still a highly demanding task for UTFAs with passive prosthesis (Eskridge et al., 2019). Therefore, a better understanding of biomechanics and energetics during running for UTFAs with RSPs is expected to aid the establishment of running gait rehabilitation, as well as further the development of spring-loaded prosthetic components.

Mechanical energy fluctuation of the body center of mass (COM) is a useful analytical approach to identify fundamental human gait mechanisms (Biewener, 2006). During locomotion, the powers of the mechanical actions acting on the body is related to the instantaneous energy fluctuations. Among them, the external mechanical work done to maintain the motion of the COM relative to the surroundings associated with the mechanical energy fluctuation of the COM (Willems et al., 1995). When individuals without amputation run at a constant speed on level ground, the mechanical energy of the COM is absorbed during the negative work phase and restored during the subsequent positive work phase (Margaria, 1968; Cavagna et al., 1976). To maintain a symmetrical bouncing gait at each step, positive work is required to replace the lost mechanical energy using additional muscular work with the energy expended (Margaria, 1968; Cavagna et al., 1976). Consequently, the net external mechanical work (*∆W*_ext_), which is defined as the difference between negative and positive works, becomes zero at each step. Furthermore, in terms of the mechanical energy fluctuations during the stance phase, the musculoskeletal system (such as muscle and tendon) of individuals without amputation can be compared with a frictionless bouncing mechanism represented by a simple spring-mass system (Blickhan, 1989). Considering the spring-like leg features in UTFAs wearing passive RSPs, the underlying mechanism of prosthetic running could be described by the mechanical energy fluctuations and the external mechanical work of the COM. However, as reviewed by Hadj-Moussa et al. (2022), no study investigated the external mechanical work during running in UTFAs.

The aim of the present study was to investigate external mechanical work at different running speeds for UTFAs wearing RSPs. According to previous studies, passive prosthetic devices cannot generate mechanical power during the stance phase of running (Brüggemann et al., 2008; Nolan, 2008; Beck et al., 2016). Furthermore, when compared to the unaffected limb, the affected limb of UTFAs suffers from muscle weakness due to atrophy of the residual limb (Sherk et al., 2010). Therefore, we hypothesized that UTFAs would perform asymmetric mechanical work between limbs, where *∆W*_ext_ is negative in the affected limb but positive in the unaffected limb at a given running speed.

## 2 Materials and Methods

### 2.1 Participants

Eight runners (6 male and 2 female) with unilateral transfemoral amputation participated in the experiment and ran with their own prescribed RSPs and prosthetic knee joints (Table 1). The including criteria for participants were as follows: 1) no neuromuscular disorders and orthopedic problems, 2) Functional Classification Level of K-4 and being able to run without external supports, 3) having a competitive athletic experience of the 1000-m sprint. All participants regularly trained between 2 and 6 days per week at the time of the experiment. Each participant ran with their own prescribed RSPs and prosthetic knee joints (Table 1). Prior to the experiment, all participants provided written informed consent. The experiment protocol was approved by the local ethics committee, and the experiment was conducted in accordance with the guidelines set out in the Declaration of Helsinki (1983).

**TABLE 1:** Subject characteristics.

Subject	Sex	Age (years)	Height (m)	Mass (kg)	Time since amputation (years)	Cause of amputation	RSP model and category of stiffness	Prosthetic knee joint	100 m PR (s)	100% speed (m s^−1^)
1	M	26	1.75	66.0	5	Trauma	Sprinter 1E90 (cat.3)	3S80	14.08	7.10
2	M	17	1.77	84.0	3	Congenital	Sprinter 1E90 (cat.4)	3S80	14.45	6.92
3	F	29	1.64	62.3	12	Trauma	Runner 1E91 (cat.4)	3S80	14.61	6.84
4	M	26	1.71	63.3	4	Trauma	Runner 1E91 (cat.3)	3S80	16.02	6.24
5	M	24	1.60	60.0	6	Trauma	KATANA-β (hard)	3S80	16.13	6.20
6	M	54	1.70	65.8	31	Trauma	KATANA-β (medium)	3S80	16.25	6.15
7	M	23	1.68	55.7	20	Cancer	Sprinter 1E90 (cat.3)	3S80	16.81	5.95
8	F	19	1.56	58.9	5	Trauma	Runner 1E91 (cat.3)	3S80	16.86	5.93
Mean		27	1.68	64.5	11				15.65	6.42
SD		11	0.07	8.1	9				1.03	0.43

Demographic and anthropometric data, time since amputation, cause of amputation, running-specific prosthesis (RSP) model, and category of stiffness, prosthetic knee model, 100-m personal record (PR), and corresponding 100% speed for each subject.

### 2.2 Experimental Procedures

**FIGURE 1:**
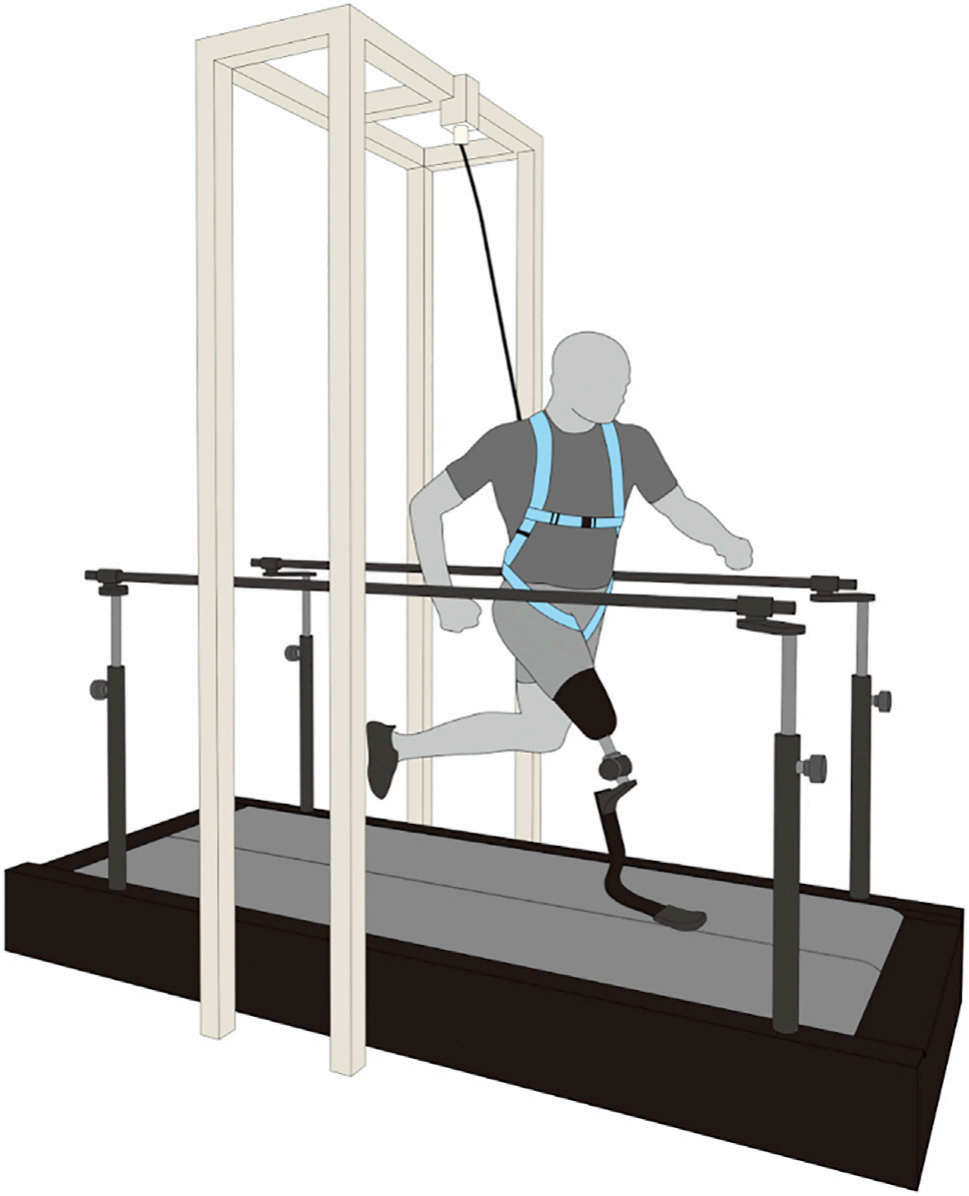
A schematic representation of the experimental setup. Eight sprinters using running-specific prosthesis ran on an instrumented split-belt treadmill at incremental speeds (30, 40, 50, 60, 70, and 80% of the average speed of his/her 100-m personal records. A safety harness was used to prevent participants from falling during experiments.

Prior to data collection, we asked each participant to walk and run on an instrumented split-belt treadmill (Figure 1; FTMH-1244WA, Tec Gihan, Kyoto, and Japan) for at least 5 min as a minimal familiarization period for running on the treadmill (Zeni and Higginson, 2010; Sakata et al., 2020a). During the familiarization period, all participants experienced six running speeds (30, 40, 50, 60, 70, and 80% of their average speed). In our study, the 100% speed for each participant was defined as the average speed of their 100-m personal record in official competitions (Table 1). The participants then started a series of trials at 30% speed, and the running speed for each subsequent trial was increased by 10% until the participants reached the 80% speed. For each running speed, the participants performed a single trial and ran for less than 20 s on the treadmill. The treadmill belt speed was constantly accelerated up to the target running speed, at an acceleration of 0.84 m s^−2^ (Hobara et al., 2019; Sakata et al., 2020a). The average running speeds for each trial of 30, 40, 50, 60, 70, and 80% were 1.92 ± 0.13 m s^−1^, 2.56 ± 0.19 m s^−1^, 3.19 ± 0.23 m s^−1^, 3.83 ± 0.26 m s^−1^, 4.47 ± 0.31 m s^−1^, and 5.10 ± 0.36 m s^−1^, respectively. Between each trial, the participants rested for as long as necessary to minimize the effects of fatigue. In addition, a safety harness was used to prevent the participants from falling; however, it was kept moderately slack to ensure the participants were running naturally (Figure 1).

### 2.3 Data Collections and Analysis

The mediolateral, anteroposterior, and vertical components of the ground reaction force (GRF) were recorded using two under belt force platforms (TF-40120-CL and TF-40120-CR; Tec Gihan, Kyoto, Japan) at a sampling frequency of 1,000 Hz. The GRF data were filtered using a fourth-order zero-lag low-pass Butterworth filter, with a cutoff frequency of 25 Hz (Kram et al., 1998; Clark and Weyand, 2014). To measure the contact of the foot on the treadmill belt, the touchdown and toe-off were identified from the filtered vertical GRF data with a threshold of 25 N (Werkhausen et al., 2019).

In general, the mediolateral GRF component during running was smaller than the anteroposterior (*F*_ap_) and vertical (*F*_v_) GRF components (Wannop et al., 2012). Furthermore, previous studies have also observed this trend in both unilateral transtibial (Baum et al., 2016) and transfemoral amputees (Makimoto et al., 2017). Consequently, the work done to sustain the mediolateral movement of the COM was negligible compared to that for the movement in other directions for UTFAs. Thus, in this study, we focused on the work done in the anteroposterior and vertical directions at the affected and unaffected limb’s steps.

In the present study, we analyzed ten consecutive steps and averaged the five steps of each limb to determine representative values at each of the six different running speeds. The computational methods of analyzing the mechanical energy and work of the COM have been published previously (Cavagna, 1975; Schepens et al., 1998; Gosseye et al., 2010), and hence, we provide a brief explanation of this analysis technique. The acceleration, velocity, and displacement of the COM were calculated using the total GRF, and which is the sum of the GRF data in the left and right force platforms. Because the air resistance is negligible, the accelerations in the anteroposterior (*a*_ap_) and vertical (*a*_v_) directions are calculated as follows:

$$\begin{array}{c} a_{\mathrm{ap}}=\frac{F_{\mathrm{ap}}}{m} \end{array},$$ (1)

$$\;\begin{array}{c} and\;\;\;\;a_{v}=\frac{F_{v}-mg}{m} \end{array},$$ (2)

where *m* is the body mass, and *g* is the acceleration due to gravity.

**FIGURE 2:**
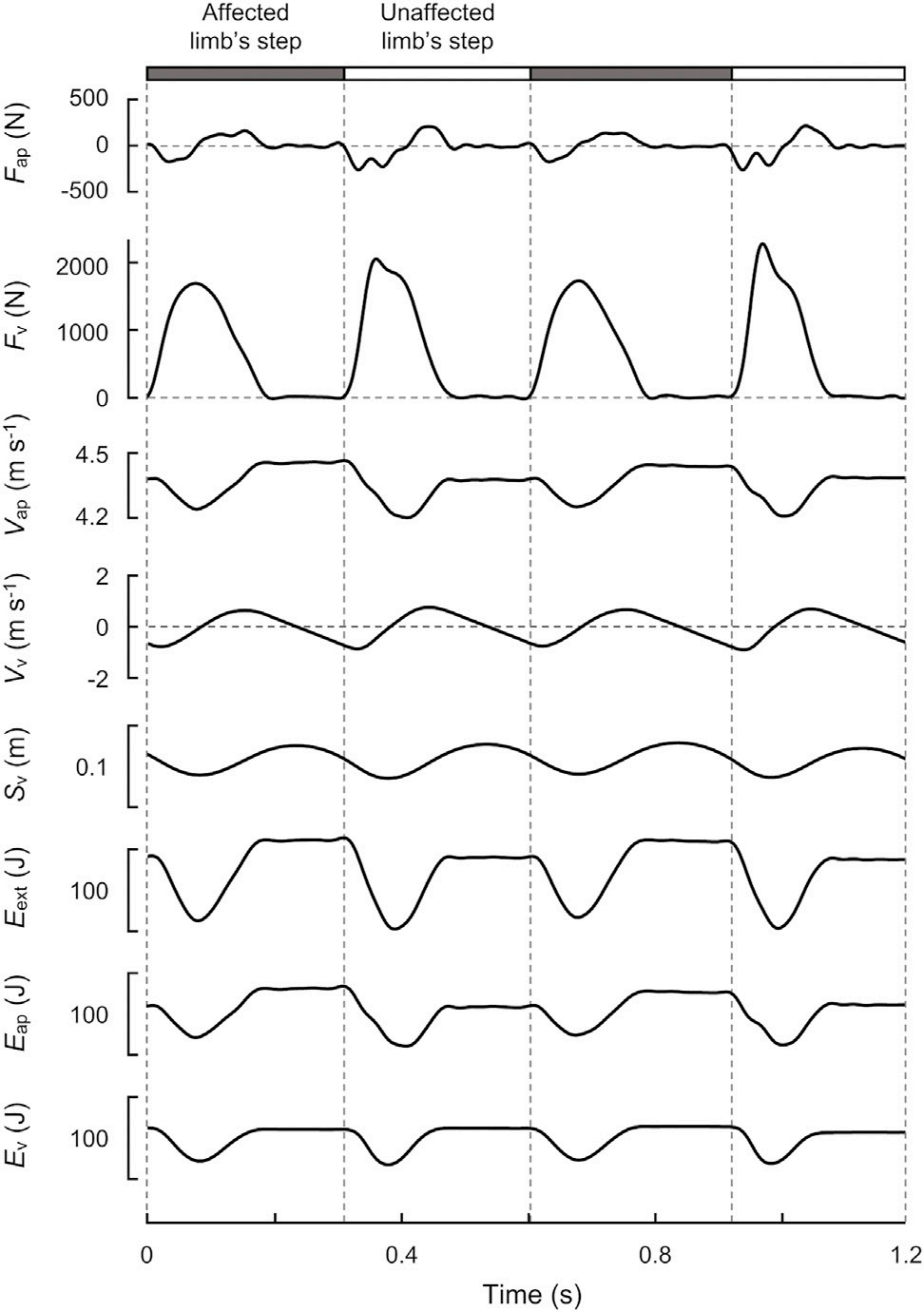
Time-course profiles of GRF, velocity, displacement, and energy of the COM over two strides at 70% speed (4.36 m s^−1^) for a representative male runner with unilateral transfemoral amputation (height: 1.71 m, mass: 63.3 kg). The gray and white bars indicate the affected and unaffected limb’s steps, respectively. From top to bottom, anteroposterior GRF (*F*_ap_), vertical GRF (*F*_v_), anteroposterior velocity (*V*_ap_), vertical velocity (*V*_v_), displacement (S_v_), external mechanical energy (*E*_ext_), anteroposterior mechanical energy (*E*_ap_), and vertical mechanical energy (*E*_v_) of the COM are shown.

The values of *a*_ap_ and *a*_v_ were time-integrated numerically to determine the velocity in the anteroposterior (*V*_ap_) and vertical (*V*_v_) directions of the COM with an integration constant (Schepens et al., 1998). In the anteroposterior direction, the participant ran at average constant speeds on the treadmill; therefore, the integration constants of *V*_ap_ were calculated using the assumption that they were equal to the average speed of the treadmill belt over a stride at each speed. In the vertical direction, the integration constants of *V*_v_ were set to zero, assuming that the COM height would be constant between the initial and end of each stride during level running (Cavagna, 1975; Schepens et al., 1998; Gosseye et al., 2010; Mesquita et al., 2020). Finally, the vertical displacement of the COM (*S*_v_) was calculated as the time-integration of *V*_v_ using an integration constant. The upper five panels of Figure 2 show a typical example of *F*_ap_, *F*_v_, *V*_ap_, *V*_v_, and *S*_v_ traced over two strides.

The external mechanical work is necessary to maintain the motion of the COM relative to the surroundings (Willems et al., 1995), associating with the fluctuation of the mechanical energy of the COM (*E*_ext_). In mechanics, this energy is composed of the kinetic and potential energies of the COM. Therefore, *E*_ext_ was computed as the sum of the energies due to its motion in the anteroposterior (*E*_ap_) and vertical (*E*_v_) directions, as follows:

$$\begin{array}{c} E_{\mathrm{ext}}=E_{\mathrm{ap}}+E_{v}, \end{array}$$ (3)

where

$$\begin{array}{c} E_{\mathrm{ap}}=\frac{1}{2}mV_{\mathrm{ap}}^{2}, \end{array}$$ (4)

$$\begin{array}{c} E_{v}=mgS_{v}+\frac{1}{2}mV_{v}^{2}. \end{array}$$ (5)

**FIGURE 3:**
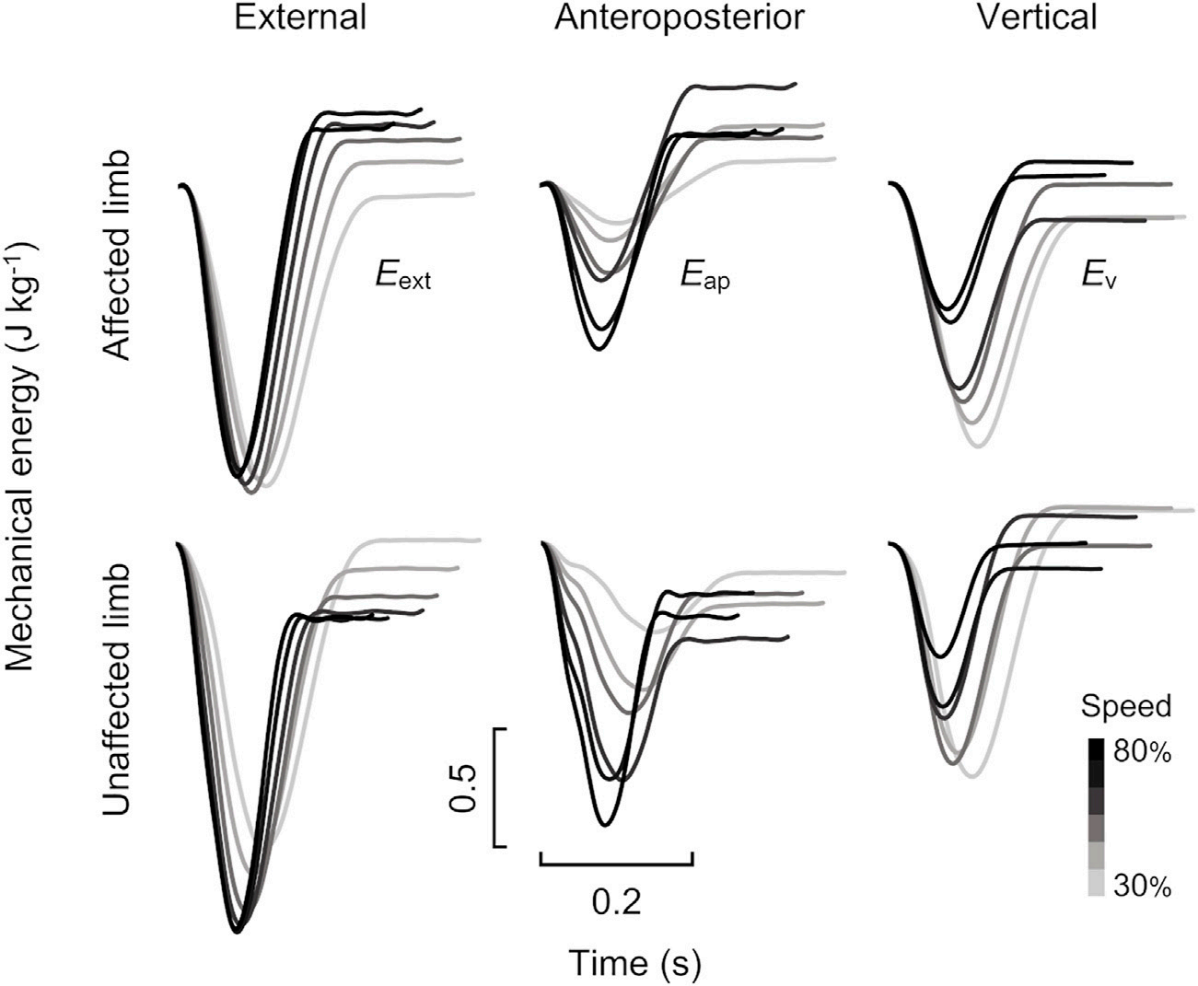
Time-course profiles of mechanical energy traces for affected **(top)** and unaffected **(bottom)** limbs for the male runner with unilateral transfemoral amputation (same runner as shown in Figure 2). The left, middle, and right panels show external (*E*_ext_), anteroposterior (*E*_ap_), and vertical (*E*_v_) mechanical energy curves, respectively. Grayscale in each panel indicates variations in running speed from 30 to 80%.

The time-course profiles of the *E*_ext_, *E*_ap_, and *E*_v_ curves at six different speeds are shown in Figure 3. Then it is possible to relate the instantaneous variation of energy to the power of the mechanical actions acting on the body and therefore the variation of the energy on a given interval of the time to the work of this same actions. In the present study, the net mechanical works per step (*∆W*_ext_, *∆W*_ap_, and ∆*W*_v_) were calculated as the difference between the *E*_ext_, *E*_ap_, and *E*_v_ curves of the initial and end of stance phases. Furthermore, *∆W*_ext_, *∆W*_ap_, and ∆*W*_v_ represent the difference between the negative (*W*_ext_^−^, *W*_ap_^−^, and *W*_v_^−^) and positive work per step (*W*_ext_^+^, *W*_ap_^+^, and *W*_v_^+^). The negative and positive work done in each direction were computed as the absolute value of the decrements and increments of the *E*_ext_, *E*_ap_, and *E*_v_ curves, respectively. Net, negative, and positive mechanical works of the COM were normalized to each participant’s body mass in the unit of J kg^−1^.

In this study, we used a parameter called *Recovery* to detect the underlying gait mechanism based on energy fluctuations. During level running, *E*_ap_ and *E*_v_ do not interchange but are simultaneously taken up and released by the muscles at each bouncing step (Cavagna et al., 1976). Specifically, these energies are in phase (Cavagna et al., 1976). The amount of energy exchange between *E*_ap_ and *E*_v_ can be expressed as the *Recovery* (%), computed as (Cavagna et al., 1976; Dewolf et al., 2016; Mesquita et al., 2020):

$$\begin{array}{c} \mathrm{Recovery}=\frac{\left|W_{\mathrm{ap}}^{+}\right|+\left|W_{\mathrm{ap}}^{-}\right|+\left|W_{v}^{+}\right|+\left|W_{v}^{-}\right|-\left(\left|W_{\mathrm{ext}}^{+}\right|+\left|W_{\mathrm{ext}}^{-}\right|\right)}{\left|W_{\mathrm{ap}}^{+}\right|+\left|W_{\mathrm{ap}}^{-}\right|+\left|W_{v}^{+}\right|+\left|W_{v}^{-}\right|}\times 100, \end{array}$$ (6)

where *Recovery* = 0% indicates that the *E*_ap_ and *E*_v_ curves are perfectly in phase. In a frictionless bouncing mechanism, *Recovery* is ideally equal to 0%.

### 2.4 Statistical Analysis

The Shapiro–Wilk test was used to determine whether the data distributions violated the normality assumption. If the data were normally distributed, the two-way repeated-measures ANOVA with two factors, limb (two levels) and speed (six levels), was used to compare the variables between the affected, and unaffected limbs across the six running speeds. To assess the assumptions of variance, Mauchly’s test of sphericity was performed using all ANOVA results. A Greenhouse–Geisser correction was performed to adjust the degree of freedom if an assumption was found to be violated, while a Bonferroni post hoc multiple comparison was performed if significant main effects or interactions were observed. For each ANOVA result, a partial eta-squared (η_p_^2^) value was calculated as a measure of the size of effect, where small was defined as 0.01 < η_p_^2^ < 0.06, moderate was defined as 0.06 < η_p_^2^ = 0.14, and large was defined as η_p_^2^ > 0.14 (Cohen, 1988). In contrast, if the data were not normally distributed (*p* < 0.05), the nonparametric Friedman test and Wilcoxon rank sum test were performed. When a significant main effect of speed was observed in the Friedman test, the Wilcoxon rank sum test with the Bonferroni correction was used for post hoc comparisons. These post hoc comparisons of the speed and limb were carried out by adjusting the alpha levels of 0.003 (= 0.05/15) and 0.008 (= 0.05/6), respectively. The effect size in non-parametric tests was computed using the *r* value (0.1 < *r* < 0.3, small; 0.3 < *r* < 0.5, moderate; *r* > 0.5, large) and the *Z* value. The equation to convert the *Z* value into the *r* value is as follows: *r* = $Z/\sqrt{N}$ , where *N* is the number of total observations (Field, 2009). Statistical significance was defined as *p* < 0.05 for all statistical tests except for the post hoc comparisons in the non-parametric tests. All statistical analyses were performed using SPSS for Windows Version 26 (IBM, Armonk, NY, United States).

## 3 Results

### 3.1 External Mechanical Work

**FIGURE 4:**
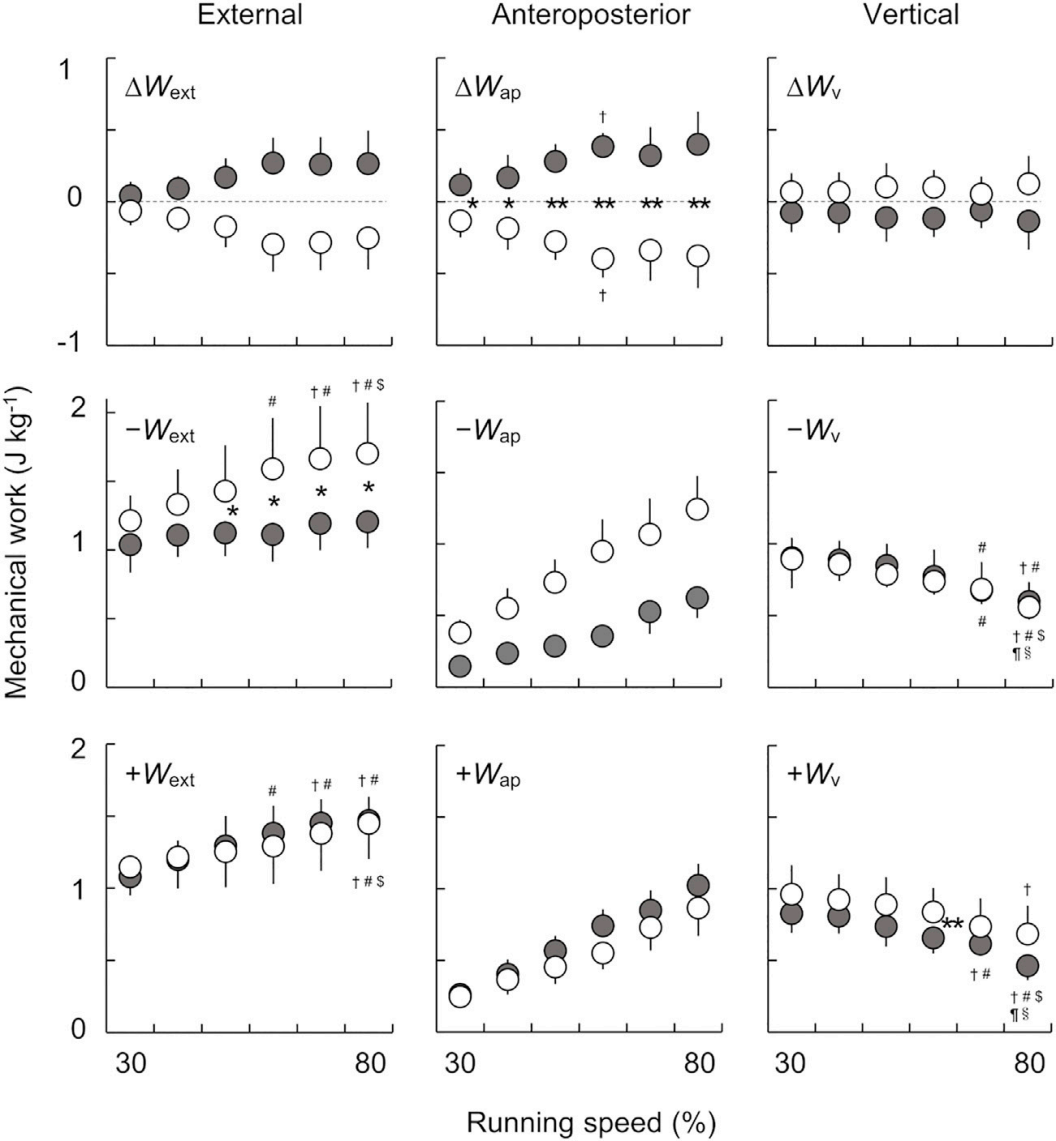
The net, negative, and positive mechanical works per step in the external **(left)**, anteroposterior **(middle)**, and vertical **(right)** components across six running speeds. Gray and white circles indicate the mean values of eight runners for the affected and unaffected limbs, respectively. The error bars represent 1 SD. The asterisks (*, **) indicate significant differences between the limbs at each speed, at *p* < 0.05 and *p* < 0.01, respectively. Dagger (†), sharp (#), dollar ($), pilcrow (¶), and section (§) symbols indicate significant differences from 30, 40, 50, 60, and 70% speeds at *p* < 0.05, respectively.

Figure 4 shows the net, negative, and positive mechanical work per step in the external, anteroposterior, and vertical components for both the affected and unaffected limbs across six running speeds. There were significant main effects of speed on *∆W*_ext_ for both the affected (*χ* ^*2*^ (5) = 18.214, *p* < 0.05) and unaffected limbs (*χ* ^*2*^ (5) = 19.571, *p* < 0.05). However, there were no significant differences between the speeds for both limbs in the post hoc analysis. The Wilcoxon rank sum test revealed that there were no significant differences in *∆W*_ext_ between the affected and unaffected limbs at each tested speed. However, *∆W*_ext_ in the affected limb tended to be greater than that in the unaffected limb across a range of speeds, where the effect sizes were either large or moderate for all comparisons (30%: *Z* = −1.260, *p* = 0.208, and *r* = −0.315; 40–60%, 80%: *Z* = −2.100, *p* = 0.036, and *r* = −0.525; 70%: *Z* = −2.380, *p* = 0.017, and *r* = −0.595).

We found significant main effects of speed (*F*_(1.99, 13.90)_ = 15.653, *p* < 0.001, and η_p_^2^ = 0.691) and limb (*F*_(1.00, 7.00)_ = 7.738, *p* = 0.027, and η_p_^2^ = 0.525) on *W*_ext_^−^. Furthermore, there was a significant interaction between the speed and limb (*F*_(1.24, 8.65)_ = 5.772, *p* < 0.05, and η_p_^2^ = 0.452). The post hoc analysis revealed that *W*_ext_^−^ in the unaffected limb significantly increased with speed, but this was not the case for the affected limb. Consequently, the magnitude of the differences in *W*_ext_^−^ between the affected and unaffected limbs increased with running speeds.

There was a significant main effect of speed on *W*_ext_^+^ (*F*_(2.04, 14.25)_ = 15.607, *p* < 0.001, and η_p_^2^ = 0.690) but not of limb (*F*_(1.00, 7.00)_ = 0.129, *p* = 0.730, and η_p_^2^ = 0.018). However, a significant interaction between the speed and limb on *W*_ext_^+^ (*F*_(5.00, 35.00)_ = 2.629, *p* < 0.05, and η_p_^2^ = 0.273) was found. Although the *W*_ext_^+^ in both limbs significantly increased with increasing speed, there was no significant difference between the limbs over all speeds.

### 3.2 Anteroposterior Mechanical Work

While there was no significant main effect of speed on *∆W*_ap_ (*F*_(5.00, 35.00)_ = 0.729, *p* = 0.606, and η_p_^2^ = 0.094), there were significant main effects of limb (*F*_(1.00, 7.00)_ = 32.922, *p* < 0.001, and η_p_^2^ = 0.825) and interaction between the speed and limb on *∆W*_ap_ (*F*_(5.00, 35.00)_ = 8.148, *p* < 0.001, and η_p_^2^ = 0.538). We found significant inter-limb differences in *∆W*_ap_ at all running speeds (30 and 40%: *p* < 0.05; 50–80%: *p* < 0.01), and the differences tended to be greater when running at 30–60% speeds.

The Friedman test showed a significant main effect of speed in *W*_ap_^−^ for both the affected (*χ* ^*2*^ (5) = 37.286, *p* < 0.001) and unaffected (*χ* ^*2*^ (5) = 40.000, *p* < 0.001) limbs. However, there were no significant differences between the speeds for both limbs in the post hoc analysis. Furthermore, the *W*_ap_^−^ in the affected limb tended to be smaller than that in the unaffected limb across a range of speeds. Although the Wilcoxon rank sum test revealed no significant differences in *W*_ap_^−^ between the affected and unaffected limbs at each speed, we found that the effect sizes were large (*r* = 0.630) at all speeds (30–80%: Z = −2.521, *p* = 0.012, and r = −0.630).

There was a significant main effect of speed on *W*_ap_^+^ for both the affected (*χ* ^*2*^ (5) = 40.000, *p* < 0.001) and unaffected limbs (*χ* ^*2*^ (5) = 38.429, *p* < 0.001). However, no significant differences between the speeds were observed for both limbs. The *W*_ap_^+^ in both the affected and unaffected limbs tended to increase with speed, but there was no significant difference in *W*_ap_^+^ between the limbs across the range of speed (30%: *Z* = −0.280, *p* = 0.779, and *r* = −0.07; 40%: *Z* = −0.420, *p* = 0.674, and *r* = −0.105; 50 and 70%: *Z* = −1.820, *p* = 0.069, and *r* = −0.455; 60%: *Z* = −2.521, *p* = 0.012, and *r* = −0.630; 80%: *Z* = −1.540, *p* = 0.123, and *r* = −0.385).

### 3.3 Vertical Mechanical Work

There were no significant main effects of speed on ∆*W*_v_ for the affected (*χ* ^*2*^ (5) = 3.786, *p* = 0.581) or the unaffected limbs (*χ* ^*2*^ (5) = 2.500, *p* = 0.776). We also found that there were no significant differences in ∆*W*_v_ between limbs at each running speed. There were no significant differences in the ∆*W*_v_ between limbs at all running speeds (30 and 50%: *Z* = −1.400, *p* = 0.161, and *r* = −0.350; 40 and 70%: *Z* = −1.260, *p* = 0.208, and *r* = −0.315; 60%: *Z* = −1.820, *p* = 0.069, and *r* = −0.455; 80%: *Z* = −1.680, *p* = 0.093, and *r* = −0.420). Consequently, ∆*W*_v_ in both the affected and unaffected limbs remained nearly constant across a range of running speeds.

Statistical analysis revealed a significant main effect of speed on *W*_v_^−^ (*F*_(2.14, 14.97)_ = 20.421, *p* < 0.001, and η_p_^2^ = 0.745); however, there was no significant main effect of limb on *W*_v_^−^ (*F*_(1.00, 7.00)_ = 0.118, *p* = 0.741, and η_p_^2^ = 0.017), nor was there an interaction found between the speed and limb (*F*_(1.80, 12.58)_ = 0.425, *p* = 0.642, and η_p_^2^ = 0.057). *W*_v_^−^ significantly decreased with increasing speed for both the affected and unaffected limbs, but there was no significant difference between the limbs at each speed.

We found significant main effects of speed (*F*_(2.14, 14.98)_ = 21.368, *p* < 0.001, and η_p_^2^ = 0.753) and limb (*F*_(1.00, 7.00)_ = 6.039, *p* < 0.05, and η_p_^2^ = 0.463) on *W*_v_^+^, while there was no significant interaction effect (*F*_(5.00, 35.00)_ = 0.706, *p* = 0.623, and η_p_^2^ = 0.092). *W*_v_^+^ of both the affected and unaffected limbs significantly decreased with increasing speed. Furthermore, *W*_v_^+^ of the affected limb was significantly smaller than that of the unaffected limb at 60% speed (*p* < 0.05).

### 3.4 Energy Transduction

**FIGURE 5:**
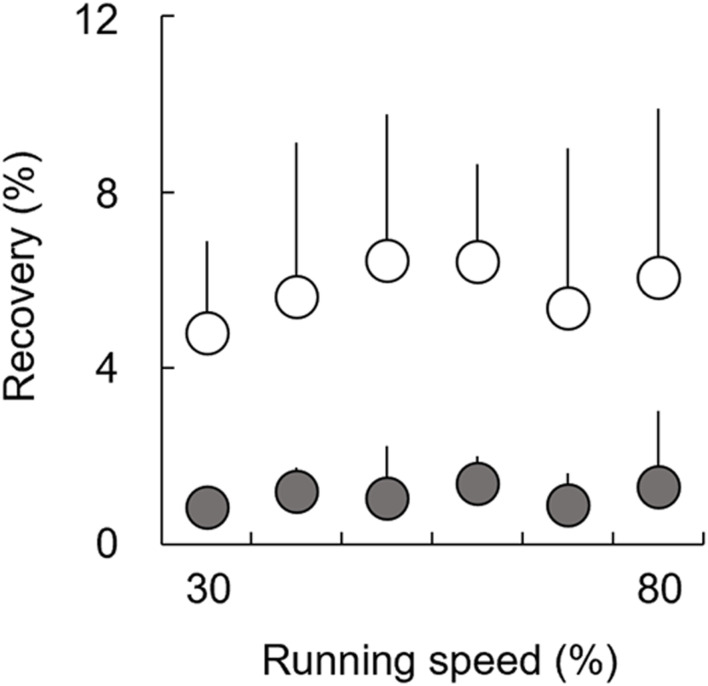
Recovery for affected (gray circles) and unaffected (white circles) limbs across six running speeds. The error bars represent 1 SD.

There were no significant main effects of speed on *Recovery* in both the affected (*χ* ^*2*^ (5) = 7.29, *p* = 0.200) and unaffected limbs (*χ* ^*2*^ (5) = 2.93, *p* = 0.711) when using the Friedman test (Figure 5). Additionally, the Wilcoxon rank sum test revealed that there were no significant differences in *Recovery* between the affected and unaffected limbs at each speed (30–80%: *Z* = −2.521, *p* = 0.012, and *r* = −0.630). However, we found that *Recovery* in the affected limb tended to be smaller than that in the unaffected limb across a range of speeds, with large effect sizes for all comparisons.

## 4 Discussion

The aim of the present study was to investigate external mechanical work at different running speeds for UTFAs wearing RSPs. As shown in Figure 4, although it did not reach statistical significance, *∆W*_ext_ in the affected limb tended to be greater than that in the unaffected limb across the six running speeds, with large or moderate effect sizes for all comparisons. Moreover, we found that *∆W*_ext_ of the affected limb was positive, while that of the unaffected limb was negative across a range of speeds. These results contradict our hypothesis that *∆W*_ext_ values in the affected and unaffected limbs are negative and positive, respectively, at different running speeds. Therefore, the results of the present study suggest that UTFAs with RSPs maintain their bouncing steps with a limb-specific strategy.

According to previous studies, passive prosthetic devices cannot generate mechanical power during running (Brüggemann et al., 2008; Nolan, 2008; Beck et al., 2016). Furthermore, the affected limb suffers muscle weakness due to atrophy of the residual limb after transfemoral amputation, where the muscle cross-sectional area in the thigh of the affected limb was approximately 28% that of the unaffected limb (Sherk et al., 2010). During locomotion, the energy variation due to the movement of the body segments done by the muscular work results in the energy variation of the COM (Cavagna et al., 1983). Therefore, the positive *∆W*_ext_ in the affected limbs indicated that UTFAs would perform additional muscular work by using residual muscles in their affected limbs and/or other whole-body muscles during stance phases. In particular, the hip muscles in the affected limb would be important for UTFAs to regain their ability to run after amputation (Nolan, 2012; Namiki et al., 2019). A previous study reported that 10-weeks training for improvement of hip strength enables UTFAs to run again after amputation (Nolan, 2012). Since the *∆W*_ext_ computed from the GRF is required to change the COM movement, the COM of UTFAs would be accelerated in the affected limb and decelerated in the unaffected limb at each bouncing step across different constant running speeds. Consequently, in terms of the external mechanical work of the COM, we found that UTFAs might rely more on the affected limb during running at different constant speeds.

While *W*_ext_^−^ of the affected limb was statistically smaller than that of the unaffected limb, there were no inter-limb differences in *W*_ext_^+^ between the affected and unaffected limbs across the range of running speeds (Figure 4). In addition, we found that there was no change of *W*_ext_^−^ with speed in the affected limb, whereas in the unaffected limb increased with the speed. The magnitude of the differences in *W*_ext_^−^ tended to be greater at higher speeds (Figure 4). However, *W*_ext_^+^ in both limbs increased with speed, with no obvious inter-limb differences. Therefore, the inter-limb difference in ∆*W*_ext_ is attributed mainly to that in *W*_ext_^−^ rather than *W*_ext_^+^ at different speeds.

During the negative work phase in non-amputee’s running, *E*_ext_ is mainly absorbed by the knee extensor muscles (Schache et al., 2015; Liew et al., 2016). However, UTFAs do not possess the biological functions of these muscles in their affected limb. Additionally, passive prosthetic knee joints in affected limbs do not have the function of energy absorption during the stance phase (Schmalz et al., 2017; Namiki et al., 2019). Therefore, *W*_ext_^−^ of the affected limb would be smaller than that of the unaffected limb and constant across a range of speeds due to the mechanical constraints of passive prostheses. These results suggest that the affected limb may exhibit less capability to absorb *E*_ext_ compared to the unaffected limb.

Conversely, during the latter half of the stance phase, individuals without amputation perform positive work to restore the lost mechanical energy in order to make *∆W*_ext_ equal zero at each step (Margaria, 1968). In individuals without amputation, the positive work done by muscles is derived from 1) the mechanical energy stored in the elastic components of the biological legs during the negative work phase and 2) additional positive muscular work by their contractile component (Cavagna and Kaneko, 1977). In terms of *Recovery* in UTFAs (Figure 5), *Recovery* in the affected and unaffected limbs was less than approximately 5% at all speeds. Previous studies have reported that *Recovery* in non-amputees is typically less than 5% at different constant running speeds (Cavagna et al., 1976; Willems et al., 1995; Dewolf et al., 2016). Runners without amputation have been considered to perform elastic behavior for energy saving to minimize muscle work by using elastic components in their biological legs, such as tendons (Cavagna et al., 1976; Cavagna and Kaneko, 1977; Werkhausen et al., 2019). The results of the present study suggest that both the affected and unaffected limbs would work as spring-like legs, similar to non-amputee legs. Surprisingly, although it did not reach statistical significance, *Recovery* in the affected limb tended to be smaller than that in the unaffected limb and was close to 0% across speeds (Figure 5). These results suggest that the affected limb with RSPs would behave as an ideal mechanical spring compared to the unaffected limb. Therefore, while some additional muscular work was required to accelerate the body during the stance phase of the affected limb, most of the *W*_ext_^+^ in the affected limb might be attributed to the mechanical energy stored through the RSPs with energy-storing capabilities. *W*_ext_^+^ in the unaffected limb might be attributed to the stored mechanical energy through elastic leg behavior that minimizes additional muscular work. Consequently, it can be assumed that there is no obvious difference in *W*_ext_^+^ between both limbs across the range of running speeds. Additionally, the present study suggests that UTFAs may perform an efficient elastic bounce of the body using energy storage and restore the capabilities of RSPs to achieve a positive *∆W*_ext_ in the affected limb, rather than a positive *∆W*_ext_ in the unaffected limb. Therefore, after lower limb amputation, the use of a spring-based passive prosthesis would be essential for UTFAs to regain running ability.

When comparing the effects of speed on *∆W*_ext_, the post hoc analysis did not show significant differences between speeds for both limbs, but there was a main effect of speed observed for both limbs. These results suggest that the inter-limb difference in *∆W*_ext_ tends to be greater at higher speeds (Figure 4). At higher running speeds (60–80% speeds), there were no obvious changes in *∆W*_ext_ for both limbs (Figure 4), indicating that the amount of stored and restored mechanical energy might reach a plateau at middle running speeds. Additionally, the specific value of ∆*W*_ext_ is dependent on the imbalance between *∆W*_ap_ and *∆W*_v_. Significant differences were observed between both limbs in *∆W*_ap_ (Figure 4). Furthermore, *∆W*_ap_ of the affected and unaffected limbs were positive and negative, respectively, across the range of running speeds. Since the *∆W*_ap_ computed from the GRF is associated with the fluctuation of *E*_ap_, the results suggest that the kinetic energy due to anteroposterior movement of the COM in UTFAs is increased (propulsion) in the affected limb but decreased (braking) in the unaffected limb at each step to maintain constant running speeds. Our results agree with previous reports that the affected limb in UTFAs generates a more positive net anteroposterior GRF impulse (i.e., the velocity change of the COM during each stance phase) at maximal sprinting (Makimoto et al., 2017; Namiki et al., 2019) and running at a wide range of constant speeds (Sakata et al., 2020b). In contrast, there were no obvious differences in *∆W*_v_ between the limbs across all speeds (Figure 4). Therefore, the inter-limb difference in ∆*W*_ext_ is mainly due to *∆W*_ap_ rather than *∆W*_v_.

Several limitations of the present study should be considered in the interpretation of the findings. First, although the analysis of the external mechanical work of the COM using force plates is a useful approach for identifying the fundamental human gait mechanism, it does not directly consider the contributions of angular motions of the body segments, joint work, body kinematics, and muscle activation during running. In the future, further biomechanical and energetic analyses of running with passive prostheses will be needed and these analyses will help to identify the solely attribution of the affected and unaffected limbs and/or other body segments. Second, the present study was conducted using an instrumented treadmill, which is not the same to overground running. Indeed, previous studies demonstrated that the parameters derived by the GRF measured by an adequate instrumented treadmill are comparable, but not directly equivalent, to those measured during overground running (Riley et al., 2008; Kluitenberg et al., 2012). Therefore, potential differences in GRF between treadmill and overground running remain unclear. Third, due to the limited number of UTFAs who can run over a wide range of speeds, only eight UTFAs were available for the present study. Further, the variation of demographic characteristics in the UTFAs was not negligible small (Table 1), such as the age (17–54 years) and the time since amputation (3–31 years). Limited sample number and demographic variation might have potential effect on the bouncing gait mechanism with passive prostheses, so caution should be used in the interpretation and generalization of current findings. Finally, participants in the present study used their own RSPs and prosthetic knee joints (Table 1) under several prosthetic configurations, such as RSP model, RSP shape, category of stiffness, and prosthetic alignments. According to previous studies (Beck et al., 2016; Beck et al., 2017a; Beck et al., 2017b; Migliore et al., 2020; Taboga et al., 2020), prosthetic configurations could affect the running biomechanics and performance in individuals with lower limb amputation. Future work should investigate the bouncing gait mechanism in runners with passive prostheses while considering different prosthetic configurations.

In general, the simple spring-mass model has been applied to describe the bouncing mechanism in human running. Regarding *Recovery* for UTFAs (Figure 5), *Recovery* of the affected and unaffected limbs takes a lower value across the range of running speeds. To some extent, running of UTFAs also can be compared to a spring–mass system. However, the current results suggest that the spring–mass model could not be applied because *∆W*_ext_ is not equal to zero for the affected or unaffected limbs of UTFAs across the range of running speeds. Therefore, analysis of mechanical energy fluctuations revealed the necessity to rethink better biomechanical models for running of UTFAs with passive prostheses.

In summary, we observed the external mechanical work of the COM during running of UTFAs wearing passive prostheses. *∆W*_ext_ in the affected limb was positive, while that in the unaffected limb was negative across a range of speeds. These results suggest that the COM of UTFAs would be accelerated in the affected limb’s step and decelerated in the unaffected limb’s step at each bouncing step across different constant speeds. Therefore, UTFAs with passive prostheses maintain bouncing steps with a limb-specific strategy during running.

## Data Availability

The data that support the findings of this study are available from the corresponding author, Hiroaki Hobara, upon reasonable request.
